# Overexpression of DBF-Interactor Protein 6 Containing an R3H Domain Enhances Drought Tolerance in *Populus* L. (*Populus tomentosa*)

**DOI:** 10.3389/fpls.2021.601585

**Published:** 2021-02-04

**Authors:** Yang Liu, Xinzhuan Yao, Lu Zhang, Litang Lu, Renxiang Liu

**Affiliations:** ^1^College of Tobacco Science, Guizhou University, Guiyang, China; ^2^The Key Laboratory of Plant Resources Conservation and Germplasm Innovation in Mountainous Region, Ministry of Education, Institute of Agro-Bioengineering, Guizhou University, Guiyang, China; ^3^College of Tea Science, Guizhou University, Guiyang, China

**Keywords:** *NtDIP6*, POD, CAT, drought stress, RT-qPCR analysis, transgenic poplars

## Abstract

Drought is the primary disaster that endangers agricultural production, including animal husbandry, and affects the distribution, growth, yield, and quality of crops. Previous study had revealed that DIP, as a potential regulator of DBF activity, played an important role in response to drought stress in maize. In this study, a total of 67 DIPs were identified from seventeen land plants, including six tobacco DIPs (NtDIPs). *NtDIP6* gene was further selected as a candidate gene for subsequent experiments based on the phylogenetic analysis and structural analysis. The transgenic tobacco and poplar plants over-expressing *NtDIP6* gene were generated using the *Agrobacterium*- mediated method. Although there was not phenotypic difference between transgenic plants and wild-type plants under normal conditions, overexpression of the *NtDIP6* gene in transgenic tobacco and poplar plants enhanced the drought tolerance under drought treatments in comparison with the wild type. The content of antioxidant defense enzymes peroxidase (POD), catalase (CAT), and the photosynthetic rate increased in *NtDIP6-*Ox transgenic tobacco and poplar plants, while the content of malondialdehyde decreased, suggesting that the overexpression of *NtDIP6* enhances the antioxidant capacity of transgenic poplar. Furthermore, the results of qRT-PCR showed that the level of expression of drought-related response genes significantly increased in the *NtDIP6-*Ox transgenic plants. These results indicated that NtDIP6, as a positive response regulator, improves drought stress tolerance by scavenging superoxide via the accumulation of antioxidant defense enzymes.

## Introduction

As sessile organisms, the growth and development of plant is severely restricted by environmental stresses, such as drought, high salinity, and high and low temperatures. Thus, plants have evolved complex mechanisms to respond and adapt to different environmental stresses at the physiological and biochemical levels (Figueiredo et al., 2012). Among the various abiotic stresses, drought stress is the major factor that hinders the growth and development of crops throughout the world (Farooq et al., 2009). Recent studies revealed that gene expression, transcriptional regulation, and signal transduction are involved in the regulation of responses of plants to drought (Zhu et al., 2010).

DBF (Dehydration responsive element binding factor), as a transcription factor, was introduced by Kizis and Pages (2002), activates drought stress tolerance genes in many plants. Moreover, the DBF is also a part of the Apetala 2/Ethylene Response Factor (AP2/ERF) transcription factor family and induces the *rab17* (responsive to abscisic acid) gene expression under drought stress conditions (Kizis and Pages, 2002). In maize, DBF1 and DBF2 are involved in *rab17* regulation through the drought-responsive element in an ABA-dependent pathway. Xu et al. (2008) identified three new *DBF* genes in *T. aestivum* (named *TaAIDFs, T. aestivum* abiotic stress-induced DBFs) by screening a wheat cDNA library after drought treatment.

A previous study found that DBF1-interactor protein 1 (DIP1) that contained two conserced core domains (R3H and SUZ) was localized in the cytoplasm and regulates the activity of DBF1 in stress responses (Saleh et al., 2006). The R3H domain is highly conserved and widely distributed in many organisms, including eubacteria, plants, fungi, and metazoans (Saleh et al., 2006). This domain is involved in the binding of polynucleotides, including DNA, RNA, and single stranded DNA (Liepinsh et al., 2003). Moreover, the SUZ domain is a conserved RNA-binding domain found in eukaryotes and enriched in positively charged amino acids. Although Saleh et al. (2006) had revealed that DIP protein was interacted with DBF protein using yeast two-hybrid analyses, the gene function of *DIP1* has not been further evaluated.

In this study, we identified DIP family genes in tobacco and other land plants. We constructed a phylogenetic tree and performed protein and gene structure analyses. We also generated transgenic tobacco and poplar plants overexpressing *NtDIP6* under the control of the 35S–CaMV promoter to explore the phenotypic changes and drought resistance of transgenic plants compared with wild type (WT). In addition, we determined the activities of peroxidase (POD) and catalase (CAT) and the content of malondialdehyde (MDA) in transgenic tobacco and poplar plants to confirm the capacity of antioxidation. We further investigated the expression pattern of drought response genes (*PtDBF1, PtWRKY1, PtWRKY3, and PtNCED1*) using RT-qPCR. The results provide valuable information on the roles of *NtDIP6* in the regulation of drought tolerance.

## Materials and Methods

### Homolog Identification

To explore the evolution relationship of *DIP* genes in land plants, seventeen sequenced species, representing the major lineage of land plants (Supplementary Table S1), were selected and analyzed. The complete genome sequences and corresponding annotation information for seventeen land plants were downloaded from the JGI and NCBI databases. The hidden Markov model profiles of the R3H (PF01424) and SUZ (PF12752) from the Pfam database was used as the queries to search for homologous sequences in the proteome data sets. Sequences with an *E*-value of 10^–4^ were considered candidates. After removing the redundant sequences and short proteins (lengths < 100 aa), the candidates were also confirmed the presence of the R3H and SUZ domains in each candidate using the SMART databases^1^ with an *E*-value cut off 10^–10^.

### Phylogenetic Trees, Motif Distribution, and Gene Structure

The full-length amino acid sequences of DIPs from seventeen land plants were aligned using MAFFT with default parameters (Katoh and Standley, 2013). A maximum-likelihood phylogeny based on the MAFFT alignment was constructed using the PhyML software under the WAG evolution model (v. 3.0, Guindon et al., 2010). Bootstrapping with 100 replicates was used to test the reliability of trees obtained (Wang et al., 2019). A phylogenetic tree was visualized using FIGTREE^2^. MEME software was used to identify conserved motifs with the default parameters^3^. Exon/intron information for the DIP genes from six flowering plants was extracted from the corresponding genome annotation database. The data were then plotted using MapInspect software^4^.

### Construction of Plasmids and Transformation of Plants

The complete coding sequence of *NtDIP6* was amplified using primers (forward primer: 5′-CAGTTTGAGTTCCCA CATTTCC-3′ and reverse primer: 5′-CTGACCATCCA CCACATTATCC-3′) from the cDNA of *N. tabacum* and cloned into the *Hin*dIII and *Xba*I sites of the expression vector pSH737. Transgenic tobacco and poplar plants were generated by the *Agrobacterium tumefaciens*-mediated transformation of strain LBA4404 using methods described previously (Fillatti et al., 1987). The transgenic tobacco and poplar plants were planted in pots with nutrient soil in soil at 25°C under 120 μmol m^–2^ s^–1^ irradiance, 50% relative humidity and a 16 h light/8 h dark photoperiod in a growth chamber.

### Drought Stress Treatment

After 1 month of growth in Murashige and Skoog (MS) media, WT and transgenic tobacco and poplar plants constitutively overexpressing *NtDIP6* were transferred into pots as described in the previous section under a 16 h light/8 h dark photoperiod at a constant temperature of 25 °C, 50% relative humidity, and light intensity of 120 μmol photons m^–2^ s^–1^ and fertilized once a week. After 1 month, three transgenic tobacco and poplar lines (each line contained three plants) and three wild type (WT) plants were used for the drought treatment experiment. This experiment was repeated three times. Similar results were observed in all the replicates. Photos of the drought-stressed plants (one wild type plants and three transgenic lines) were taken after 2 weeks of drought treatment. The collected samples were immediately frozen in liquid nitrogen, and their RNA was extracted for subsequent experiments.

### qRT-PCR Analyses

Total RNA of transgenic poplar plants was extracted using a Quick RNA Isolation Kit (Huayueyang, Beijing, China). The total RNA was treated with DNaseI according to the manufacturer’s instructions. One microgram of total RNA of each sample was used as a template for cDNA synthesis using a SuperScript gDNA Removal cDNA Synthesis Kit (Huayueyang). For quantitative real-time reverse transcriptase–PCR (RT-qPCR), SYBR Premix Ex Tag was used on a CFX Connect Real-Time PCR system (Bio-Rad, Shanghai, China).

The qRT-PCR reactions were conducted in 96-well plates. The melting temperature of the products was determined to verify the specificity of the amplified fragments. The primers used for qRT-PCR are listed in Supplementary Table S2. The results were analyzed by the ΔΔ^CT^ method usin*g PtActin* as the reference gene. All the qRT-PCR data points were established as three biological replicates, and three technical replicates were conducted.

### Determination of Enzyme Activities

Transgenic tobacco lines, poplar lines and wild type (WT) seedlings were treated with PEG 6000 to simulate drought stress for 15 d, and the treated plant leaves (the fifth leaf from the base to the top) were used to detect the activities of POD and CAT enzymes using a kit (Beijing Suolaibao Biotechnology Co., Ltd., Beijing, China). Each lines contained three individuals.

## Results

### Genome-Wide Identification and Phylogenetic Analysis of DIP Proteins in *N. tabacum*

Two conserved domains [R3H (PF01424) and SUZ (PF12752)] of the DIP family proteins were downloaded from the Pfam database (v. 33.1) and used as queries to search the seventeen land plant genomes using the HMMER package (Supplementary Table S1). A total of 67 proteins were considered as candidates, and those proteins contained R3H and SUZ domains. Basic information of the DIP proteins was listed in Table 1, including the protein ID in NCBI, chromosomal distribution, sequence length, isoelectric point, and protein molecular mass, and gene length. In tobacco, *NtDIP3* and *NtDIP4* were on chromosome 11; *NtDIP1* was on chromosome 24, and *NtDIP2*, 5, 6 were on three scaffolds (Nitab4.5_0001437, Nitab4.5_0009285, and Nitab4.5_0011086), respectively. The length of DIP proteins ranged from 281 to 589 amino acids; the isoelectric point was between 8.3 and 9.27, and the molecular mass was 31.19∼57.44 kDa (Table 1).

**TABLE 1:**
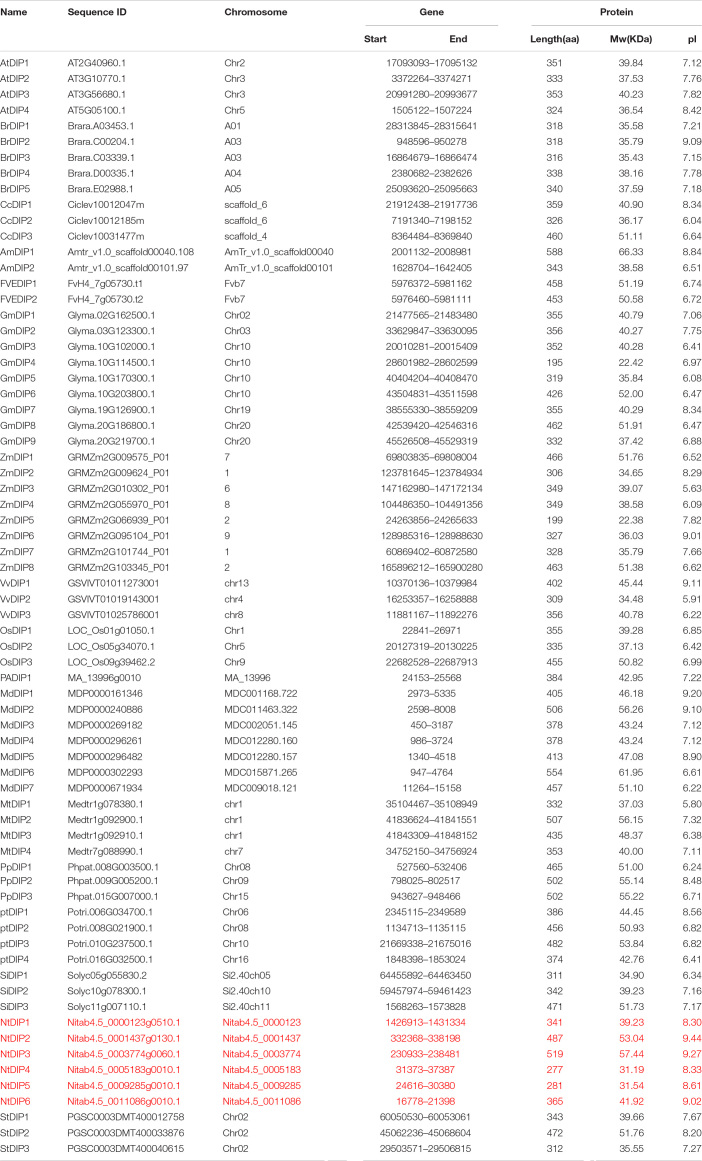
The information of DIP family genes in seventeen land plants.

*The information of NtDIP proteins was marked by red color.*

**FIGURE 1:**
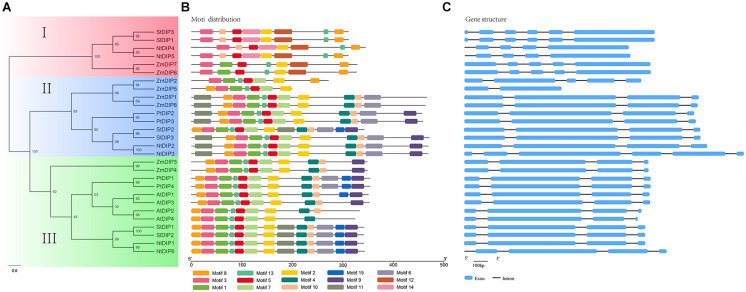
Maximum-likelihood phylogeny **(Left)**, protein motif **(Middle)**, and gene structure **(Right)** analyses of the DIP proteins from tobacco and five other flowering plants. **Left:** the phylogeny was constructed based on the amino acid sequences of full-length DIP proteins with 100 bootstrapping replicates. Rectangular color blocks represent different groups **(Left)**. **Middle:** the motif analysis was performed using PhyML. **Right:** The gene structure analysis was conducted based on genome annotation. Blue boxes represent exons, and Dashes represent introns.

To study the deeper relationships among the DIP family members in tobacco, a maximum-likelihood phylogenetic tree was constructed based on the MAFFT alignment of these DIP proteins from seventeen land plants. According to the phylogeny, the DIP proteins could be divided into three groups: I, II, and III (Supplementary Figure S1). The group I was mainly composed of the members of flowering plants except *A. trichopoda*, group II includes almost all the species investigated except the members of f *A. thaliana* and *B. rapa*. The group III contained the members of twelve species investigated except the members of *A. thaliana, B. rapa, P. trichocarpa, F. vesca*, and *P. abies*. This result suggested that the members of groups II and III was more ancient than the members of goup I. In addition, the topological structure of the phylogenetic tree is further verified by the motif analysis of DIPs (Supplementary Figure S2). In addition, the two tobacco genes (NtDIP1 and NtDIP6) were clustered with the maize DIP1 and with putative proteins [AtDIP3 (AtNP_191227) and AtDIP1 (AtNP_565947)] from *Arabidopsis* (Figure 1 and Supplementary Figure S1).

### Motif and Gene Structures of the *DIP* Genes

The phylogenetic analysis, protein motif, and gene structures of the DIP family genes from six representative flowering plants were further analyzed (Figure 1). A total of 15 conserved motifs (motifs 1–15) were identified using the MEME suite (Figure 1B and Supplementary Figure S3). Motifs 1, 3, 5, 8, and 13 correspond to the R3H domain, and motifs 2 and 7 correspond to the SUZ domain and have been identified in nearly all DIP proteins. Motif 12 and 14 were specifically distributed in group I, while motif 5 was primarily distributed in group III. Motifs 4, 6, 7, 9, and 11 were commonly distributed in groups II and III. In addition, motif 10 was only present in the DIP family proteins in dicots. The analysis of gene structure indicated that 21 (75%) of the 28 DIP family genes possess three introns (Figure 1C). The average number of introns per intron-containing *DIP* genes was 3.25. Only one gene contained a single intron, while the others contained four to five introns. The *DIP* genes in same group displayed a similar gene structure (Figure 1).

### The Overexpression of *NtDIP6* in Tobacco and Poplar Enhanced Drought and Oxidative Stress Tolerance

**FIGURE 2:**
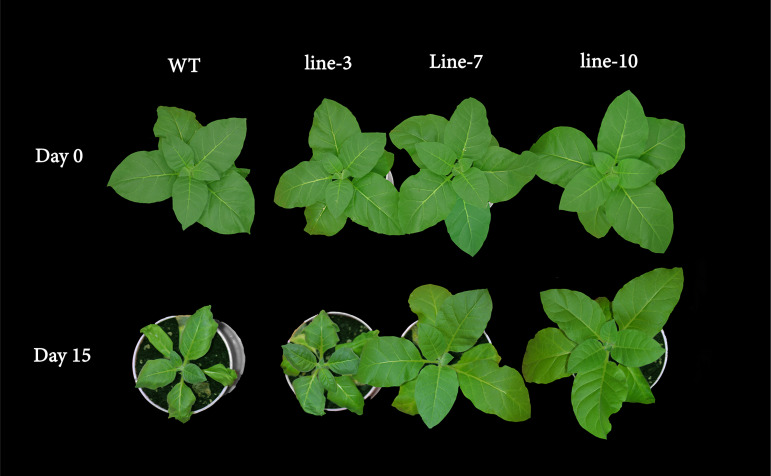
Phenotypic differences between the WT and transgenic tobacco plants under drought stress. The WT (Left) and *35S:NtDIP6* overexpressing (L3, 7 and 10, right) tobacco plants after 2 weeks of drought treatment.

**FIGURE 3:**
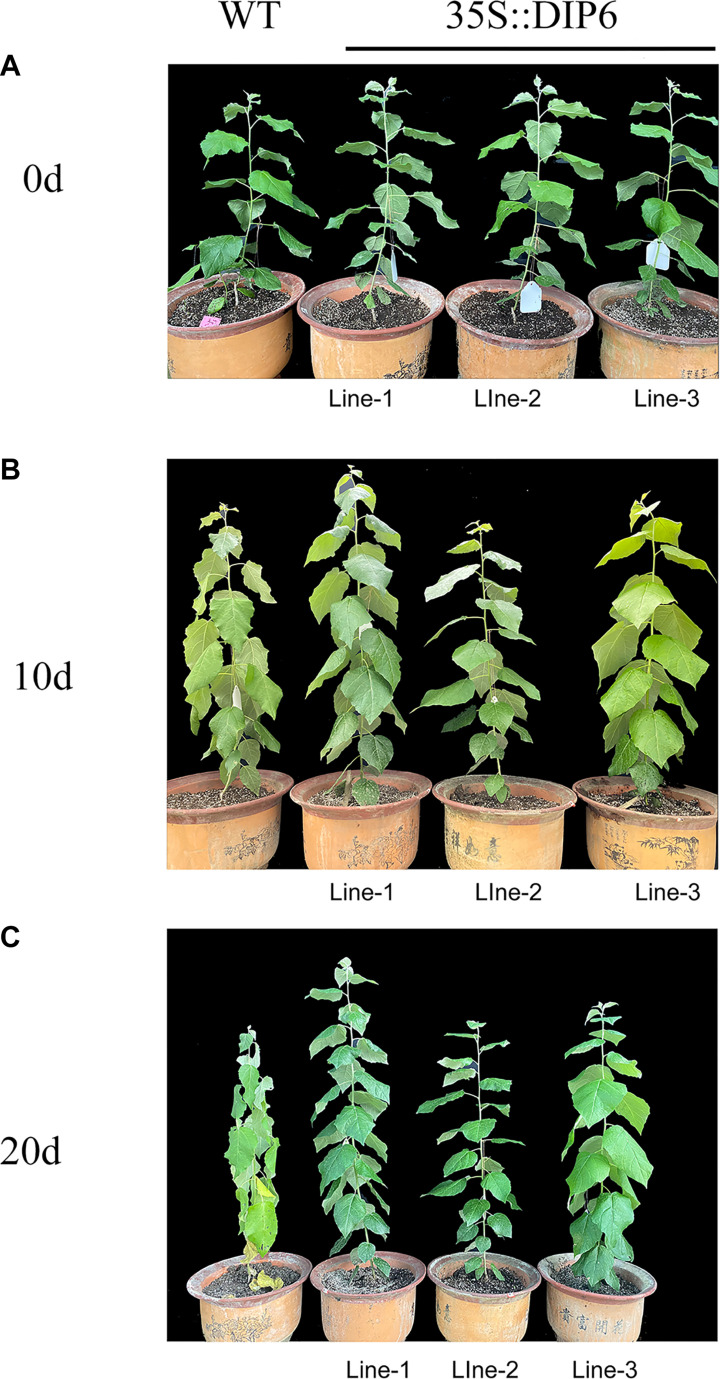
Phenotypic differences between the WT and transgenic poplar plants under drought stress. **(A)** The WT (left) and *35S:NtDIP6* overexpressing (L1–3, right) poplar plants before drought treatment, **(B)** 10 days after the stop of watering, and **(C)** 20 days after the stop of watering.

We further investigate the expression pattern of *NtDIP1* and *NtDIP6* genes under drought stress, found that the expression level of these two genes was increased after drought treatment, and the expression level of *NtDIP6* is 1.3 times the expression level of *NtDIP1* (Supplementary Figure S4). Thus, we select NtDIP6 as a candidate gene for functional verification. Transgenic tobacco and poplar plants was generated overexpressing *NtDIP6* gene under the control of 35S promoter after PCR detection and GUS staining (Supplementary Figure S5). Transgenic plants overexpressing *NtDIP6* displayed the same phenotypes in comparison with the WT plants under the normal conditions (data not shown). Drought stress is one of the limiting factors that inhibits the yields of crops throughout the world. Among various techniques that enhance the drought resistance of plants, the use of genetic modification technology has proven to be promising (Hervé and Serraj, 2009). Thus, the drought stress tolerance was examined on WT and transgenic plants after 15 days of drought treatment (Figures 2, 3). Compared with the initial treatment, the growth and development of wild type tobacco was severely inhibited after 15 days of drought treatment, and the leaves displayed some degree of wilting, while there is no significant change in the transgenic tobacco plants (Figure 2). In addition, the leaves of wild type poplars had begun to wilt after 10 days of drought treatment in comparison with initial treatment, while no change was found in the leaves of transgenic poplar plants. Although the WT and transgenic poplars wilted, the wilted phenotype of wild type after drought treatment for 20 days was more severe than that of transgenic poplar plants (Figure 3).

**FIGURE 4:**
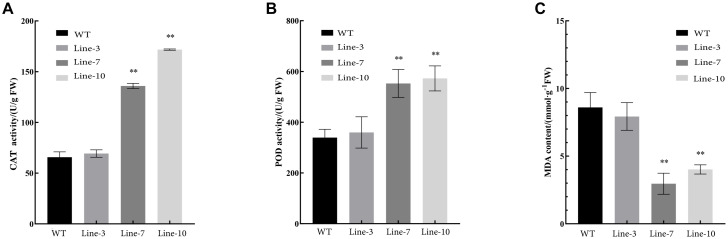
The activities of POD and CAT and the content of MDA in transgenic and wild type tobacco plants subjected to drought. **(A,B)** Higher activities of catalase (CAT) and peroxidase (POD) were observed in the leaves of transgenic tobaccos than in the leaves of WT plants after PEG treatment. **(C)** A lower content of malondialdehyde (MDA) in transgenic tobacco than in the leaves in WT after PEG treatment. Asterisks above the error bars indicate significant differences between transgenic tobacco lines and WT (^∗^*p* < 0.05; ^∗∗^*p* < 0.01). WT, wild type; Lines 3, 7, and 10, transgenic poplar lines 3, 7, and 10.

**FIGURE 5:**
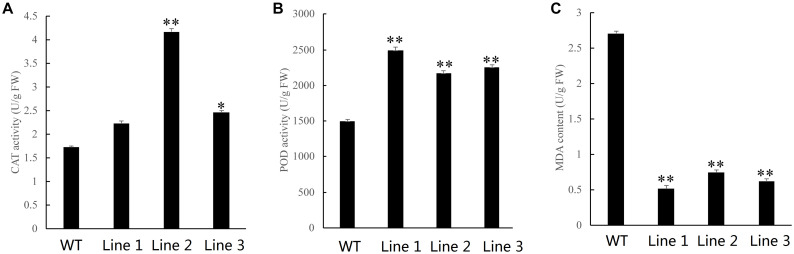
The activities of POD and CAT and the content of MDA in transgenic and wild type poplar plants subjected to drought. **(A,B)** Higher activities of catalase (CAT) and peroxidase (POD) were observed in the leaves of transgenic poplars than in the leaves of WT plants after PEG treatment. **(C)** A lower content of malondialdehyde (MDA) in transgenic poplars than in the leaves in WT after PEG treatment. Asterisks above the error bars indicate significant differences between transgenic poplar lines and WT (*p < 0.05; **p < 0.01). WT, wild type; Lines 1–3, transgenic poplar lines 1, 2, and 3.

Oxidative stress tolerance is a basis of tolerance to many abiotic stresses, such as drought and extreme temperatures. To further compare the difference in antioxidant capacity between wild type and transgenic tobacco and poplar plants overexpressing *NtDIP6*, we measured the content of MDA and the activities of POD and CAT in WT and transgenic tobacco and poplar plants after PEG treatment. Compared with wild type tobacco, the content of CAT in transgenic tobacco lines 7 and 10 was increased by 2.07 and 2.61 times (Figure 4A), and the content of POD in transgenic tobacco lines 7 and 10 was increased by 1.63 and 1.69 times in comparison after PEG treatment, respectively (Figure 4B). In addition, the content of MDA in transgenic tobacco lines 7 and 10 was decreased by 0.34 and 0.47, respectively (Figure 4C). The content of CAT and POD in the transgenic poplars were higher than those in the WT plants (Figures 5A,B). In addition, the content of MDA in the transgenic poplar was significantly lower than that in the wild type (Figure 5C). These results indicated that the transgenic plants were more tolerant to drought than the wild type.

### Improved Photosynthetic Capacity in Transgenic Poplars That Overexpress *NtDIP6*

**FIGURE 6:**
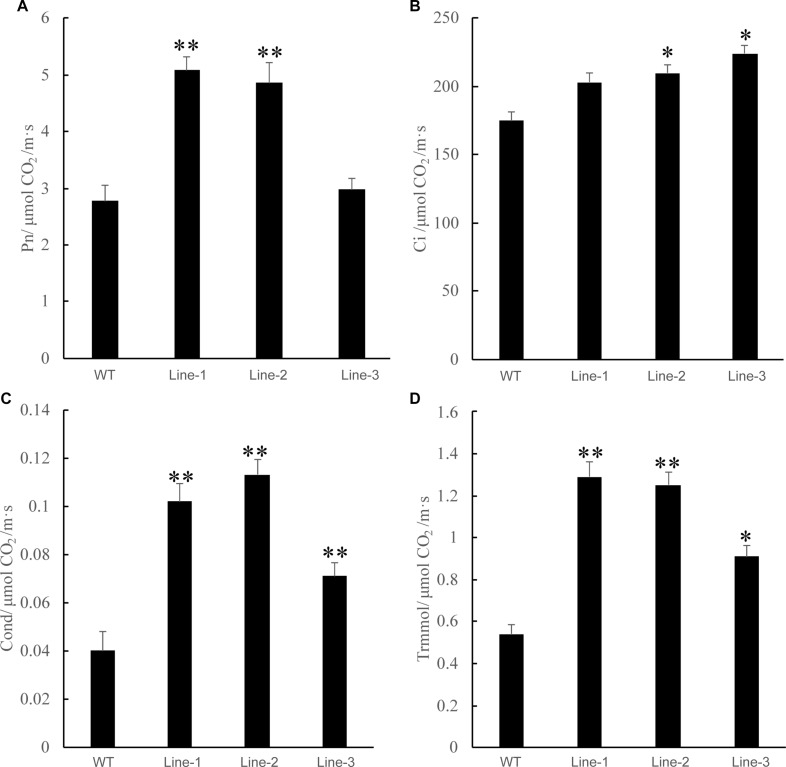
Analysis of the net photosynthetic rate and the intercellular CO_2_ concentration (Ci) in WT and transgenic poplars (Lines 1–3) in response to drought stress. **(A)** Net photosynthetic rate. **(B)** Carbon dioxide concentration. **(C)** Cond stomatal conductance. **(D)** Trmmol transpiration rate. Asterisks above the error bars indicate significant differences between trangenic poplar lines and WT (^∗^*p* < 0.05; ^∗∗^*p* < 0.01). WT, wild type; Lines 1–3, transgenic poplar lines 1, 2, and 3.

Photosynthesis is the complex process by which plants use solar energy to produce glucose from carbon dioxide and water. Improving the water use efficiency of plants under drought stress is one of the ways to enhance plant drought resistance. Previous studies have shown that a high photosynthetic capability decreases the stomatal conductance and closes stomata, which increase the intrinsic and instantaneous water-use efficiency under short term drought stress (Guo et al., 2014; Bian et al., 2019). We compared the net photosynthetic rate, CO_2_ concentration, stomatal conductance, and transpiration rate between transgenic poplars and the WT and found that these values in the transgenic plants were higher than those of the WT poplars after 2 weeks of drought treatment. The results of photosynthetic analysis showed that this photosynthetic rate was higher in the transgenic poplar compared with that in the WT poplar (average over this time period: 10:00–17:00 sunny day) (Figure 6).

### Effects of the Overexpression of *NtDIP6* on the Levels of Transcripts of Drought-Related Response Genes

**FIGURE 7:**
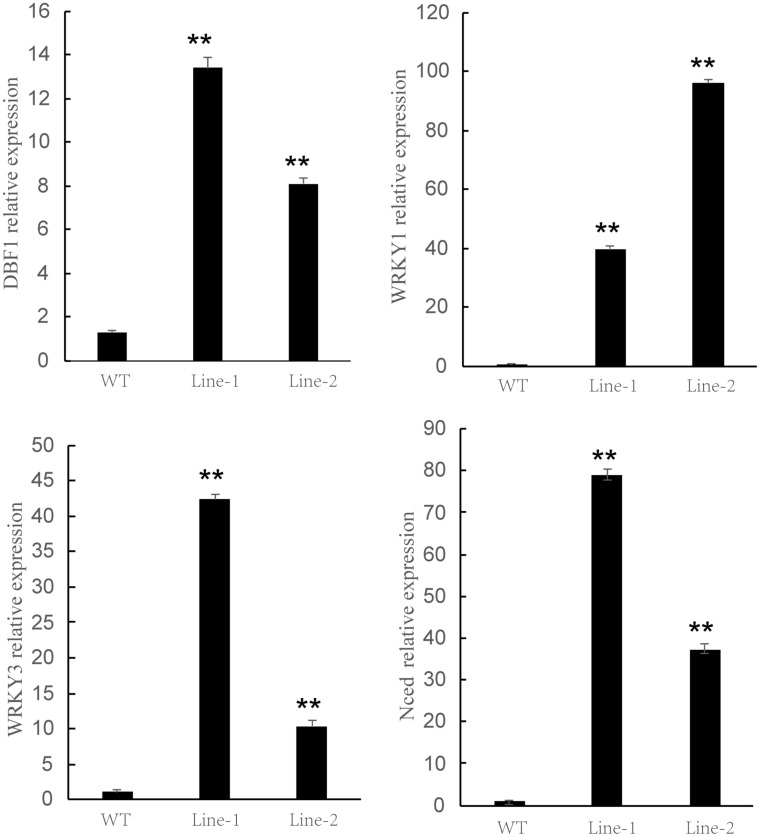
The expression pattern of drought response genes in *NtDIP6-*Ox transgenic poplars subjected to drought treatment. The expression levels relative to Actin were measured by quantitative RT-qPCR. Three biological replicates and three technical replicates were obtained for each data point. Asterisks above the error bars indicate significant differences between trangenic poplar lines and WT (^∗∗^*p* < 0.01). WT, wild type; Lines 1, 2, transgenic poplar lines 1, 2.

To further verify the drought resistance of transgenic plants, we identified the expression level of homologous sequences of four representative drought-responsive genes derived from the previous studies (Saleh et al., 2006; Zhu et al., 2016; Hwang et al., 2018; Rongrong et al., 2018), named *PtDBF1, PtWRKY1, PtWRKY3*, and *PtNCED*. The expression level of four genes was investigated in both the transgenic and WT plants under drought treatment using qRT-PCR (Figure 7). All the selected drought-related genes were highly expressed in *NtDIP6*-overexpressed poplar compared with the WT poplar, and the transgenic lines seemed to possess a higher level of selected drought-related genes. This suggested that *NtDIP6* was involved in the regulation of the expression of genes related to drought stress.

## Discussion

Plants, as sessile organisms, cannot escape from the environmental stresses that can negatively impact their survival, development, and productivity. Plants have evolved complex mechanisms at the physiological and biochemical levels to adapt to different stresses (Figueiredo et al., 2012). Drought stress is one of the major environmental factors that affects the growth and development of plants. Previous studies have revealed that TFs are involved in binding the promoters of drought response genes to enhance the tolerance of plants. This is the first study to identify DIP family genes in seventeen land plants. A total of 67 DIP genes have been identified and divided into three groups based on phylogenetic, motif, and gene structure analyses (Supplementary Figure S1 and Table 1). Furthermore, two tobacco *NtDIP* genes (*NtDIP1* and *NtDIP6*) and *Arabidopsis DIP3* identified were clustered together and distributed in group III, suggesting that these genes may have similar functions (Figure 1).

The transgenic plants overexpressing *NtDIP6* gene under the control of 35S promoter were generated. No significant difference in phenotype between transgenic plants and wild type was detected under normal conditions. Overexpression of *NtDIP6* enhanced the drought resistance of transgenic plants (Figures 2, 3). These results were consistent with a previous study that DIP1 is a potential regulator of DBF1 activity in stress responses (Saleh et al., 2006). The DBF1 and DBF2 are involved in *rab17* regulation through the drought-responsive element in an abscisic acid-dependent pathway in plants (Kizis and Pages, 2002; Xu et al., 2008). Oxidative stress tolerance is a basis of tolerance to many abiotic stresses, such as drought and extreme temperatures. The accumulation of reactive oxygen species, such as O^–2^ and H_2_O_2_, results in cell membrane peroxidation and degreasing, and increased permeability and ion outflow (Liu et al., 2005; Yao et al., 2017). Endogenous protective enzyme systems in the plants removed reactive oxygen free radicals and avoided the toxicity of free radicals to enhance the adaptability of plants. POD and CAT, as the protective enzyme systems in plants, are mostly involved in repairing the damage to cell membrane and signal transduction that results from drought stress (Wang et al., 2005; Liu et al., 2016). MDA, one of the final products of lipid peroxidation of plant cell membranes, which represents the degree of damage to plants: an increase in the content of MDA results in greater damage to plant cells (Pinhero et al., 1997; Shu et al., 2011). *NtDIP6*-overexpressing tobacco and poplar plants exhibited enhanced drought resistance with higher antioxidant enzyme activities (CAT and POD), a lower MDA content (Figures 4, 5), which is consistent with the findings of the previous studies (Saleh et al., 2006; Ning et al., 2017). In addition, NtDIP6-overexpressing poplar improved photosynthetic capacity with high net photosynthetic rate, CO2 concentration, stomatal conductance, and transpiration rate. Several studies have reported the existence of a positive correlation between photosynthetic efficiency maintenance and tolerance to drought stress in plants amended with compost and/or inoculated with AMF/PGPR (Wu et al., 2006; Sandhya et al., 2010; Tartoura, 2010; Abd El-Mageed et al., 2018, 2019; Duo et al., 2018; Khosravi Shakib et al., 2019). In this study, the expression level of four drought-responsive genes (*PtDBF1, PtWRKY1, PtWRKY3*, and *PtNCED1*) were highly expressed in *NtDIP6*-overexpressed poplar compared with the WT poplar. Previous studies had revealed that four genes (*DBF1, WRKY1, WRKY3*, and *NCED*) plays a positive regulatory role in drought stress (Saleh et al., 2006; Zhu et al., 2016; Hwang et al., 2018; Rongrong et al., 2018), which is similarly to our results of qRT-PCR.

In summary, we first identified *NtDIP* genes and randomly screened *NtDIP6* as a candidate gene for transgenic functionality. We found that transgenic plants that expressed *NtDIP6* displayed greater resistance to drought and oxidation. The level of expression of five drought-related response genes increased in *NtDIP6*-Ox transgenic poplars in comparison with the WT. Therefore, *NtDIP6* can be used as a candidate gene for the molecular breeding of drought-tolerant varieties of poplar and has potential economic value in improving drought tolerance.

## Data Availability Statement

The original contributions presented in the study are included in the article/Supplementary Material, further inquiries can be directed to the corresponding author/s.

## Author Contributions

YL and XY conceived and designed the project. XY and LZ grew the plant material and conducted the experiments. YL contributed analytical tools and analyzed data. XY wrote the manuscript. LL and RL oversaw the experiments and revised the manuscript. All the authors read and approved the manuscript.

## Conflict of Interest

The authors declare that the research was conducted in the absence of any commercial or financial relationships that could be construed as a potential conflict of interest.

## Footnotes

1 http://smart.embl-heidelberg.de/

2 http://tree.bio.ed.ac.uk/software/fifigtree/

3 http://meme-suite.org/

4 http://mapinspect.software.informer.com/

## Supplementary Material

Supplementary Figure 1 Maximum-likelihood phylogeny of the DIP proteins from seventeen land plants. The phylogeny was constructed based on the amino acid sequences of full-length DIP proteins with 100 bootstrapping replicates. Green, black and orange arcs indicate different groups of DIP proteins.

Supplementary Figure 2 The logo sequence in Supplementary Figure S1.

Supplementary Figure 3 The logo sequence in Figure 1B.

Supplementary Figure 4 The expression pattern of *NtDIP1* and *NtDIP6* after 3 h of drought treatment. The expression levels relative to Actin were measured by quantitative RT-qPCR. Three biological replicates and three technical replicates were obtained for each data point. Asterisks above the error bars indicate significant differences between control and treated tobacco plants (^∗^*p* < 0.05).

Supplementary Figure 5 Molecular analyses of *NtDIP 6* transgenic poplars. (a) Schematic map of construct. Expression of NtDIP6 is driven by the 35S promoter. (b) PCR verification of different independently regenerated transgenic lines. L1–3: different transgenic poplar lines. (c) GUS staining in leaves of NtDIP6 transgenic poplars.

Supplementary Table 1 Distribution of Land-plant *DIP* genes among every subgroup of the phylogeny in Supplementary Figure S1.

Supplementary Table 2 The primers used in this study.
