# Supplementary figures and images for: Overexpression of DBF-Interactor Protein 6 Containing an R3H Domain Enhances Drought Tolerance in Populus L. (Populus tomentosa)
Source: Front Plant Sci. 2021 Feb 4;12:601585. doi: 10.3389/fpls.2021.601585 (PMC7890038; doi:10.3389/fpls.2021.601585)

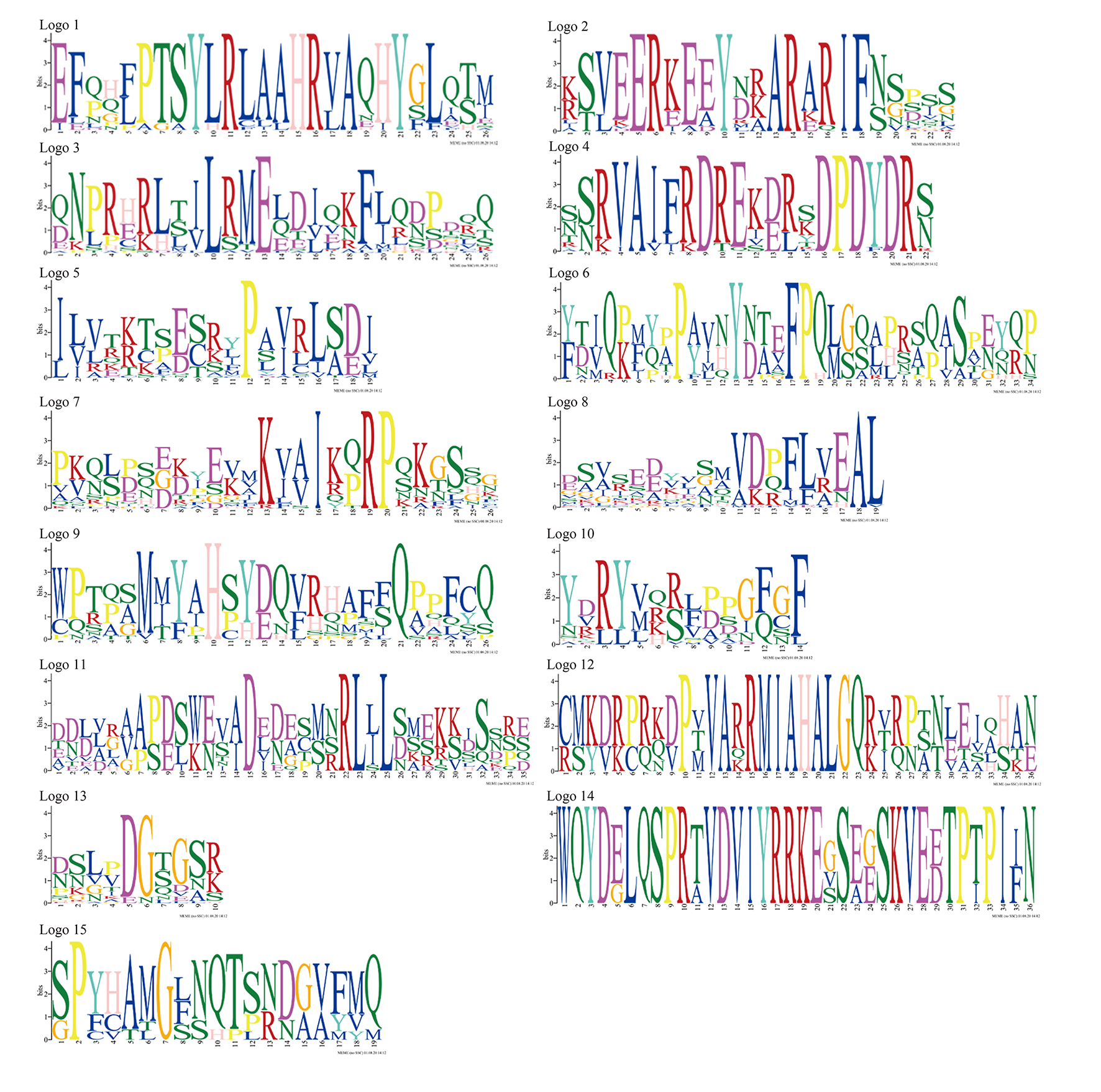

Supplement: Supplementary Figure 1 — Maximum-likelihood phylogeny of the DIP proteins from seventeen land plants. The phylogeny was constructed based on the amino acid sequences of full-length DIP proteins with 100 bootstrapping replicates. Green, black and orange arcs indicate different groups of DIP proteins. [file Image_1.TIF]

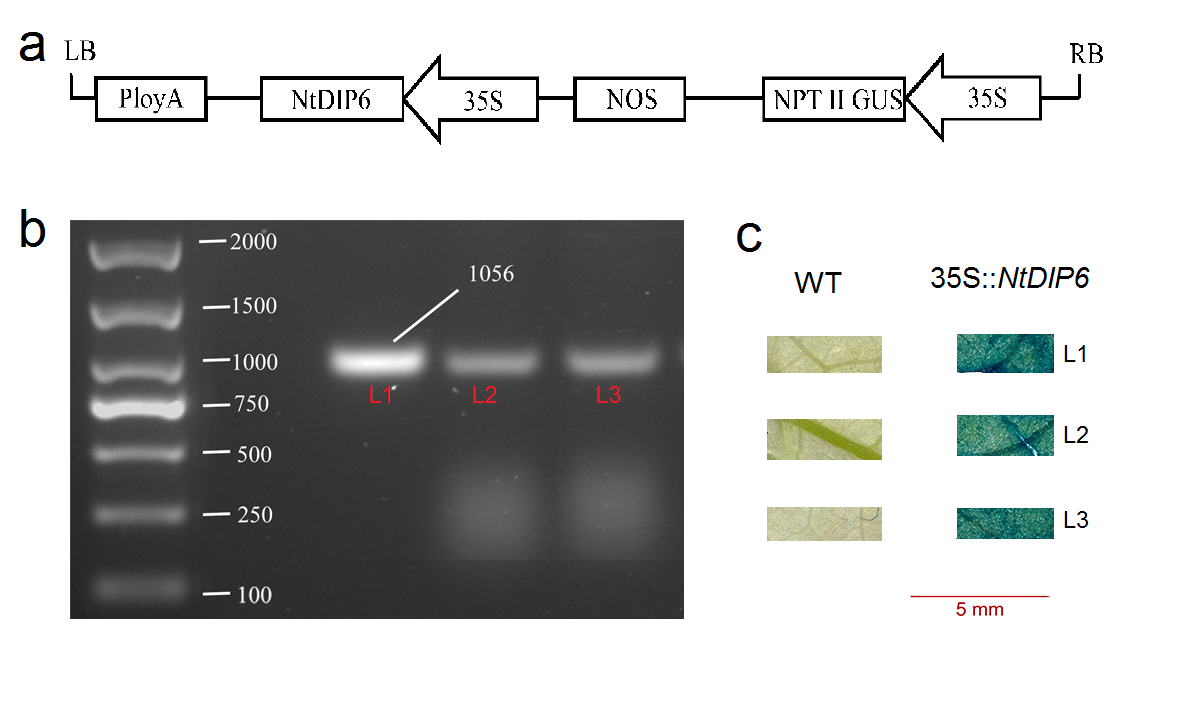

Supplement: Supplementary Figure 2 — The logo sequence in Supplementary Figure S1. [file Image_2.TIF]

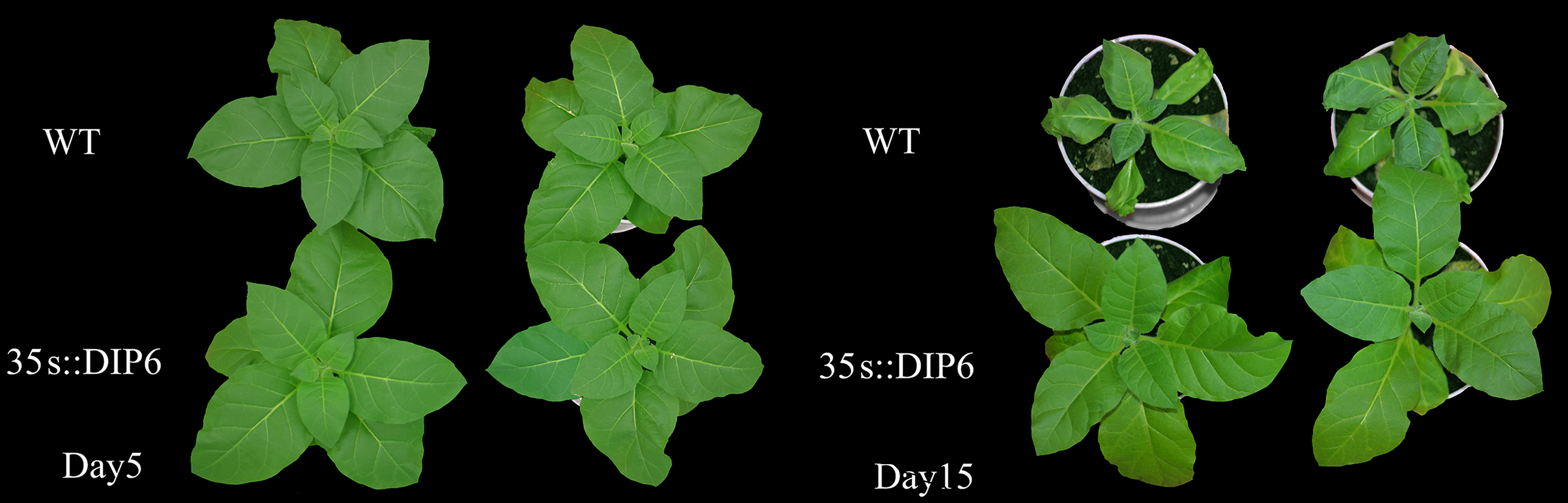

Supplement: Supplementary Figure 3 — The logo sequence in Figure 1B. [file Image_3.TIF]

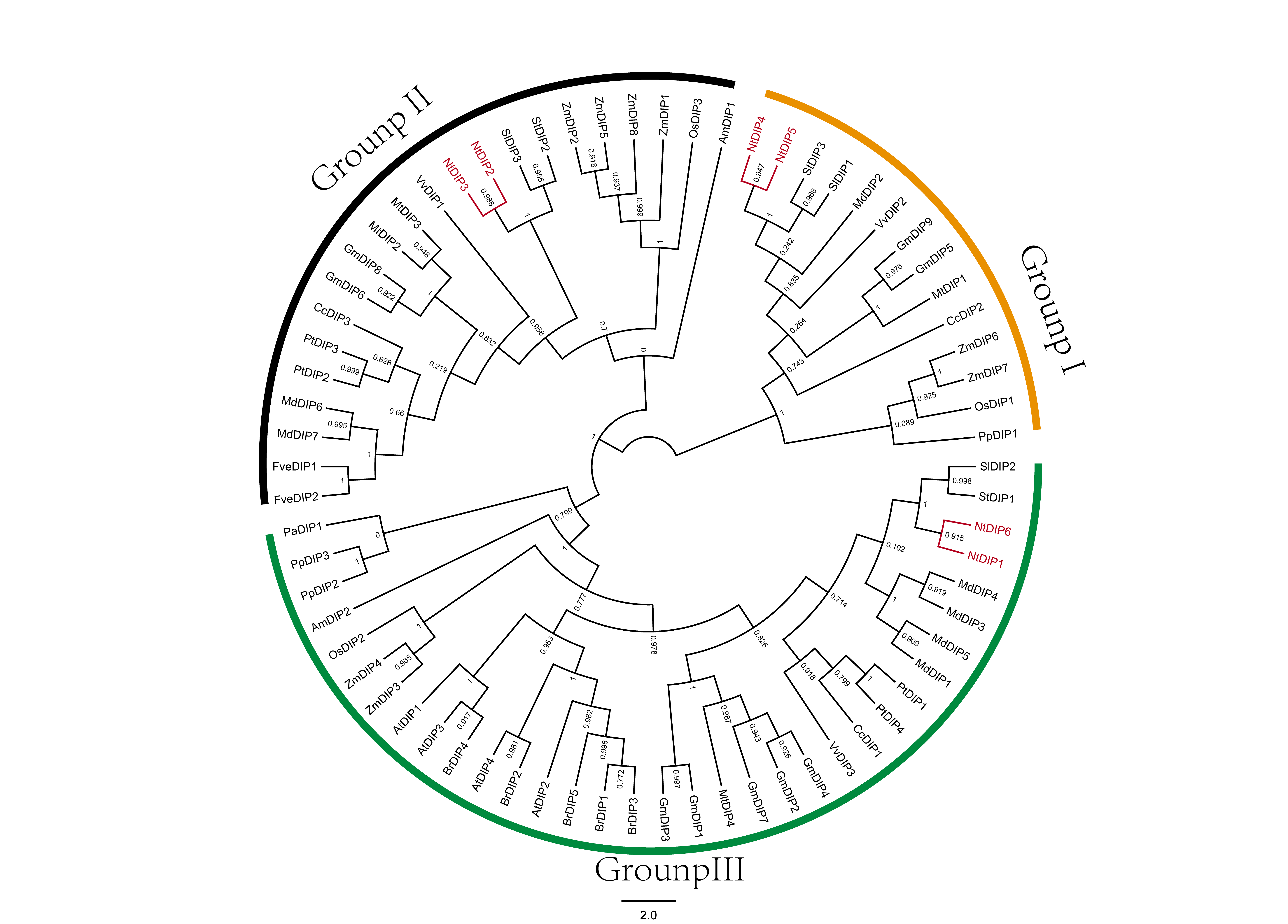

Supplement: Supplementary Figure 4 — The expression pattern of NtDIP1 and NtDIP6 after 3 h of drought treatment. The expression levels relative to Actin were measured by quantitative RT-qPCR. Three biological replicates and three technical replicates were obtained for each data point. Asterisks above the error bars indicate significant differences between control and treated tobacco plants (∗p < 0.05). [file Image_4.TIF]

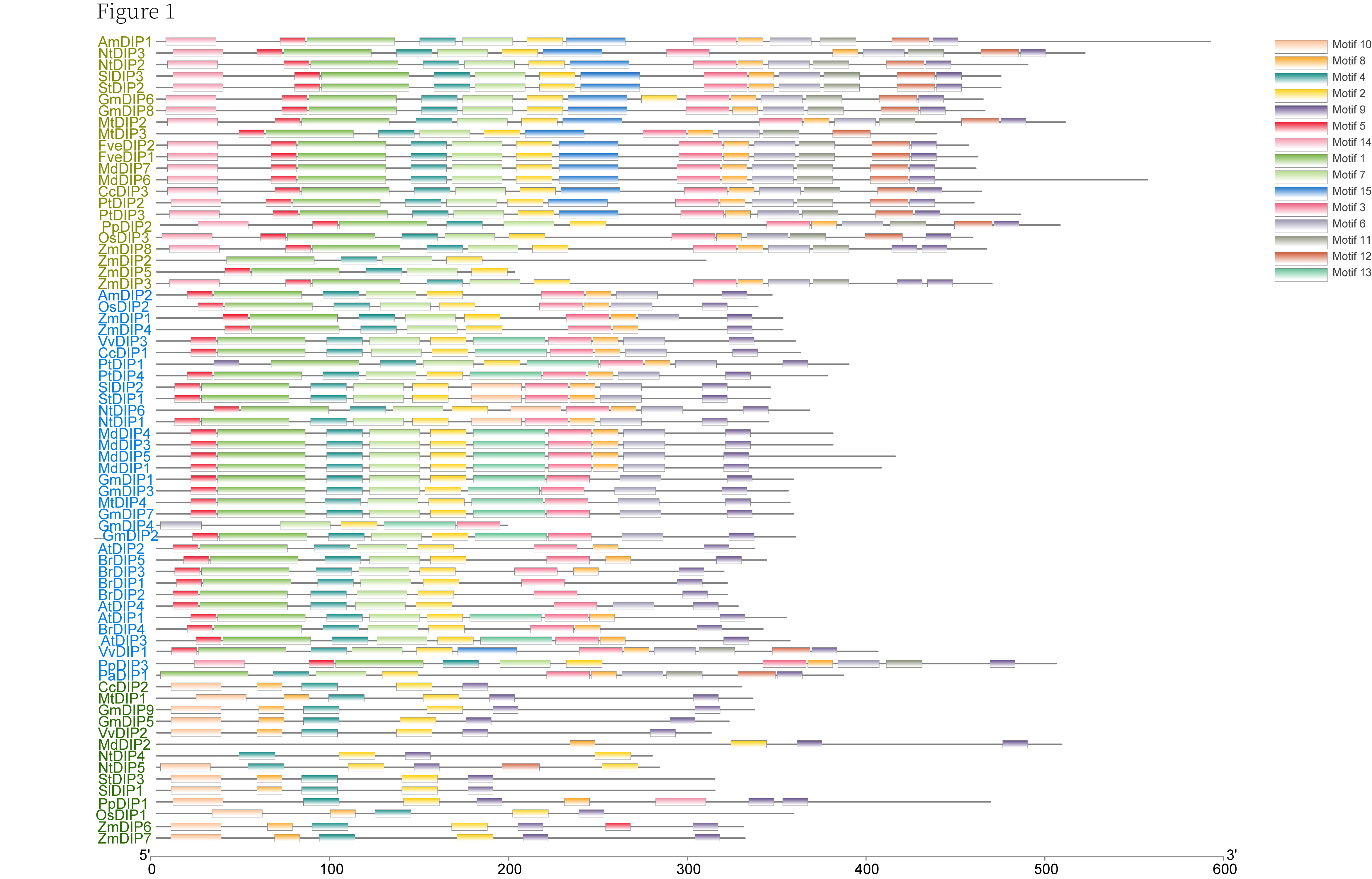

Supplement: Supplementary file 9 [file Image_8.TIF]

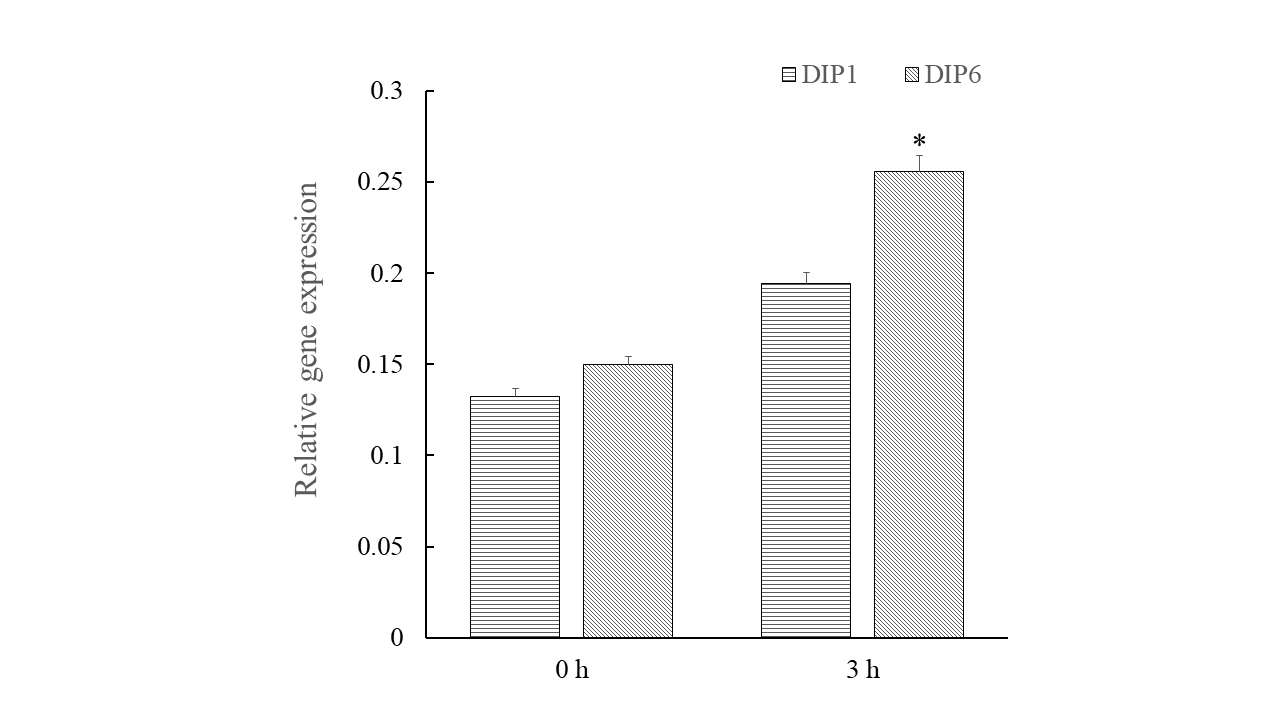

Supplement: Supplementary file 11 [file Image_10.TIF]
