# Supplementary material for: Overexpression of DBF-Interactor Protein 6 Containing an R3H Domain Enhances Drought Tolerance in Populus L. (Populus tomentosa)
Source: Front Plant Sci. 2021 Feb 4;12:601585. doi: 10.3389/fpls.2021.601585 (PMC7890038; doi:10.3389/fpls.2021.601585)
Supplement: Supplementary Table 1 — Distribution of Land-plant DIP genes among every subgroup of the phylogeny in Supplementary Figure S1. [file Data_Sheet_1.zip › Data Sheet 2.DOCX]

| GENE NAME | Abbrev | Chr.No | GENE | | | PROTEIN | | | |
| --- | --- | --- | --- | --- | --- | --- | --- | --- | --- |
|  |  |  | START | END | No. of Intron | | Length | MW (Da) | PI |
| Potri.006G034700 | PtDIP1 | Chr06 | 2345115 | 2349589 | 4 | | 364 | 40282.9 | 7.68 |
| Potri.008G021900 | PtDIP2 | Chr08 | 1134713 | 1140240 | 4 | | 468 | 50925.1 | 7 |
| Potri.010G237500 | PtDIP3 | Chr10 | 21669338 | 21675016 | 4 | | 471 | 51129.2 | 7.87 |
| Potri.016G032500 | PtDIP4 | Chr16 | 1848398 | 1853024 | 4 | | 364 | 40395.1 | 7.23 |
| Solyc05g055830 | SlDIP1 | Chr05 | 64455892 | 64463450 | 6 | | 319 | 34907.2 | 6.79 |
| Solyc10g078300 | SlDIP2 | Chr10 | 59457974 | 59461423 | 4 | | 350 | 39232.6 | 7.76 |
| Solyc11g007110 | SlDIP3 | Chr11 | 1568263 | 1573828 | 4 | | 483 | 51731.9 | 7.77 |
| Nitab4.5_0000123g0510 | NtDIP1 | Chr24 | 49222396 | 49226817 | 4 | | 350 | 39232.8 | 8.42 |
| Nitab4.5_0001437g0130 | NtDIP2 | Nitab4.5_0001437 | 332368 | 338198 | 4 | | 481 | 51791.1 | 8.58 |
| Nitab4.5_0003774g0060 | NtDIP3 | Chr11 | 33740729 | 33748277 | 6 | | 481 | 51820.1 | 8.58 |
| Nitab4.5_0005183g0010 | NtDIP4 | Chr11 | 74295971 | 74301985 | 4 | | 353 | 38765.7 | 7.9 |
| Nitab4.5_0009285g0010 | NtDIP5 | Nitab4.5_0009285 | 24616 | 30380 | 4 | | 319 | 34891.3 | 7.33 |
| Nitab4.5_0011086g0010 | NtDIP6 | Nitab4.5_0011086 | 16778 | 21398 | 4 | | 351 | 39261.8 | 8.68 |
| GRMZM2G009575 | ZmDIP1 | Chr07 | 69803835 | 69808004 | 4 | | 487 | 51759.1 | 7.01 |
| GRMZM2G009624 | ZmDIP2 | Chr01 | 123781645 | 123784934 | 4 | | 277 | 30503 | 6.17 |
| GRMZM2G010302 | ZmDIP3 | Chr06 | 147162980 | 147172134 | 4 | | 357 | 39070.3 | 5.84 |
| GRMZM2G055970 | ZmDIP4 | Chr08 | 104486350 | 104491356 | 4 | | 357 | 38574.9 | 6.52 |
| GRMZM2G066939 | ZmDIP5 | Chr02 | 24263856 | 24265633 | 2 | | 203 | 22384 | 8.24 |
| GRMZM2G095104 | ZmDIP6 | Chr09 | 128985316 | 128988630 | 5 | | 335 | 36033.2 | 9.19 |
| GRMZM2G101744 | ZmDIP7 | Chr01 | 60869402 | 60872580 | 5 | | 336 | 35787.2 | 7.86 |
| GRMZM2G103345 | ZmDIP8 | Chr02 | 165896212 | 165900280 | 4 | | 475 | 51378.5 | 7.14 |
| PGSC0003DMG400007195 | StDIP1 | Chr10 | 59434193 | 59437711 | 4 | | 350 | 39140.6 | 8.08 |
| PGSC0003DMG400019487 | StDIP2 | Chr11 | 5192651 | 5198148 | 4 | | 350 | 39140.6 | 8.08 |
| PGSC0003DMG400023471 | StDIP3 | Chr11 | 51419524 | 51425758 | 6 | | 319 | 35028.3 | 7.6 |
| AT2G40960 | AtDIP1 | Chr02 | 17095522 | 17097454 | 4 | | 361 | 39835.2 | 7.59 |
| AT3G10770 | AtDIP2 | Chr03 | 3372264 | 3374271 | 4 | | 341 | 37533.7 | 8.09 |
| AT3G56680 | AtDIP3 | Chr03 | 20991280 | 20993677 | 4 | | 363 | 40227.8 | 8.35 |
| AT5G05100 | AtDIP4 | Chr05 | 1505122 | 1507224 | 4 | | 332 | 36548.7 | 8.59 |
