# Supplementary material for: Overexpression of DBF-Interactor Protein 6 Containing an R3H Domain Enhances Drought Tolerance in Populus L. (Populus tomentosa)
Source: Front Plant Sci. 2021 Feb 4;12:601585. doi: 10.3389/fpls.2021.601585 (PMC7890038; doi:10.3389/fpls.2021.601585)
Supplement: Supplementary Table 1 — Distribution of Land-plant DIP genes among every subgroup of the phylogeny in Supplementary Figure S1. [file Data_Sheet_1.zip › Data Sheet 3.DOCX]

| **Gene Name** | **Gene ID** | **Primer sequence (5’-3’)** |
| --- | --- | --- |
| *NtDIP6* | XM_016632836.1 | CCACATTTCCCTACTTCCTACC  CCTGACCATCCACCACATTAT |
| *DPF1* | Potri.001G110700 | GAGGTTGCCTGTGCCTATTT  CTCCCTCCTGAAATCTTCCATTAC |
| *NCED* | Potri.001G393800 | GGAGGTACACAAGCACACTTAC  CCGATTTCCACTCCTTCTCATC |
| *PtWRKY1* | Potri.003G111900 | GGATGGAAGTGGCAGGATATTT  GTCTCTTCACTCTTGCTCTTGG |
| *PtWRKY2* | Potri.005G085200 | GGAGAAGACTCGGCTTATGTG  CGCGAGAGGAATCTGGTTATT |
| *HRG* | Potri.006G204300 | GGGCATAGAGTTGCGTTTAGA  GGGCTGTTCTTGACTGACTT |
| *Actin* | Potri.001G309500 | AAACTGTAATGGTCCTCCCTCCG  GCATCATCACAATCACTCTCCGA |
