# Supplementary material for: Overexpression of DBF-Interactor Protein 6 Containing an R3H Domain Enhances Drought Tolerance in Populus L. (Populus tomentosa)
Source: Front Plant Sci. 2021 Feb 4;12:601585. doi: 10.3389/fpls.2021.601585 (PMC7890038; doi:10.3389/fpls.2021.601585)
Supplement: Supplementary Table 1 — Distribution of Land-plant DIP genes among every subgroup of the phylogeny in Supplementary Figure S1. [file Data_Sheet_1.zip › Supplementary Table 1.DOCX]

| **Group** | **Family** | **Species** | **Abreve** | **I** | **II** | **III** | **Total** | **Source** |
| --- | --- | --- | --- | --- | --- | --- | --- | --- |
| Core eudicot | Brassicaceae | *Arabidopsis thaliana* | At | 4 | 0 | 0 | 4 | *jgi* |
|  | Solanaceae | *Nicotiana tabacum* | Nt | 2 | 2 | 2 | 6 | *ncbi* |
|  | Brassicaceae | *Brassica rapa* | Br | 4 | 0 | 0 | 4 | *jgi* |
|  | Rutaceae | *Citrus clementina* | Cc | 1 | 1 | 1 | 3 | *jgi* |
|  | Solanaceae | *Solanum lycopersicum* | SI | 1 | 1 | 1 | 3 | *jgi* |
|  | Solanaceae | *Solanum tuberosum* | St | 1 | 1 | 1 | 3 | *jgi* |
|  | Leguminosae | *Medicago truncatul* | Mt | 1 | 2 | 1 | 4 | *jgi* |
|  | Leguminosae | *Glycine max* | Gm | 5 | 2 | 2 | 9 | *jgi* |
|  | Rosaceae | *Fragaric vesca* | Fv | 0 | 2 | 0 | 2 | *jgi* |
|  | Rosaceae | *Malus domestica* | Md | 4 | 2 | 1 | 7 | *jgi* |
|  | Salicaceae | *Populus trichocarpa* | Pt | 2 | 2 | 0 | 4 | *jgi* |
|  | Vitaceae | *Vitis vinifara* | Vv | 1 | 1 | 1 | 3 | *jgi* |
| Monocot | Gramineae | *Zea may* | Zm | 2 | 4 | 2 | 8 | *jgi* |
|  | Gramineae | *Oryza sativa* | Os | 1 | 1 | 1 | 3 | *jgi* |
|  | Amborellaceae | *Amborella trichopoda* | Am | 0 | 1 | 1 | 2 | *jgi* |
| Gymnosperm | Pinaceae | *Picea abies* | Pa | 0 | 1 | 0 | 1 | *jgi* |
| Lower land plants | Funariaceae | *Physcomitrella patens* | Pp | 0 | 2 | 1 | 3 | *jgi* |

**Table S1 Distribution of Land-plant *DIP* genes among every subgroup of the phylogeny in Figure S1.**
